# Pharmacokinetics and Safety of Clofazimine in Children With Rifampicin-Resistant Tuberculosis

**DOI:** 10.1093/infdis/jiaf057

**Published:** 2025-02-11

**Authors:** Jennifer A Hughes, Belén P Solans, Anthony J Garcia-Prats, Heather R Draper, H Simon Schaaf, James C Nielsen, Elri Nortier, Ingrid Courtney, Megan Palmer, Louvina van der Laan, Radojka M Savic, Anneke C Hesseling

**Affiliations:** Desmond Tutu TB Centre, Department of Paediatrics and Child Health, Faculty of Medicine and Health Sciences, Stellenbosch University, Cape Town, South Africa; Department of Bioengineering and Therapeutic Sciences, Schools of Pharmacy and Medicine; UCSF Center for Tuberculosis, University of California San Francisco; Desmond Tutu TB Centre, Department of Paediatrics and Child Health, Faculty of Medicine and Health Sciences, Stellenbosch University, Cape Town, South Africa; Department of Pediatrics, School of Medicine and Public Health, University of Wisconsin, Madison; Desmond Tutu TB Centre, Department of Paediatrics and Child Health, Faculty of Medicine and Health Sciences, Stellenbosch University, Cape Town, South Africa; Desmond Tutu TB Centre, Department of Paediatrics and Child Health, Faculty of Medicine and Health Sciences, Stellenbosch University, Cape Town, South Africa; Department of Pediatrics, Hassenfeld Children's Hospital at NYU Langone, New York, New York; Desmond Tutu TB Centre, Department of Paediatrics and Child Health, Faculty of Medicine and Health Sciences, Stellenbosch University, Cape Town, South Africa; Desmond Tutu TB Centre, Department of Paediatrics and Child Health, Faculty of Medicine and Health Sciences, Stellenbosch University, Cape Town, South Africa; Desmond Tutu TB Centre, Department of Paediatrics and Child Health, Faculty of Medicine and Health Sciences, Stellenbosch University, Cape Town, South Africa; Desmond Tutu TB Centre, Department of Paediatrics and Child Health, Faculty of Medicine and Health Sciences, Stellenbosch University, Cape Town, South Africa; Department of Bioengineering and Therapeutic Sciences, Schools of Pharmacy and Medicine; UCSF Center for Tuberculosis, University of California San Francisco; Desmond Tutu TB Centre, Department of Paediatrics and Child Health, Faculty of Medicine and Health Sciences, Stellenbosch University, Cape Town, South Africa

**Keywords:** children, clofazimine, pharmacokinetics, safety, tuberculosis

## Abstract

**Background:**

We described the pharmacokinetics and safety of clofazimine in children treated for multidrug/rifampicin-resistant tuberculosis (MDR/RR-TB).

**Methods:**

Children aged <18 years were eligible. Clofazimine was administered by weight-based dosing. Sparse and semi-intensive pharmacokinetic sampling was completed at baseline and weeks 2 and 16. Clofazimine weekly area under the concentration time-curve (wAUC) was compared with the target wAUC (60.87 mg × h/L and 111.79 mg × h/L) in adults receiving clofazimine (100 mg) daily for MDR/RR-TB and leprosy, respectively. Safety monitoring included measurement of QT interval prolongation and laboratory assessment.

**Results:**

Twenty children were included: median age was 6.0 years (IQR, 1.6–14.4); 6 (30%) were male. Median clofazimine wAUC was 162.94 (IQR, 130.06–263.95), >25% higher than the target adult wAUC in adults with MDR/RR-TB (111.79; IQR, 81.9–151.9). No serious or grade ≥3 cardiac events occurred. There was a QT interval increase of 0.02 milliseconds for every 1-µg/L increase in clofazimine concentration. One severe adverse event (elevated alanine transferase) led to temporary withdrawal of clofazimine.

**Conclusions:**

The clofazimine doses used achieved substantially higher exposures in children than adults receiving standard clofazimine doses. The association of higher clofazimine exposures and QT interval prolongation may pose unnecessary risk to children, particularly in combination with other QT-prolonging drugs.

**Clinical Trials Registration:**

South African National Clinical Trials Register (https://sanctr.samrc.ac.za/; DOH-27-0620-6415).

## Teaser

We investigated the pharmacokinetics and safety of clofazimine in children routinely treated for rifampicin-resistant tuberculosis. Semi-intensive and sparse sampling was completed. Clofazimine exposures were considerably higher than protocol-specified adult targets, with a linear relationship observed between clofazimine concentrations and increasing QT interval.

Model-based estimates suggest that each year 25 000 to 32 000 children aged <15 years develop multidrug-resistant/rifampicin-resistant tuberculosis (MDR/RR-TB)—specifically, disease caused by *Mycobacterium tuberculosis* resistant to at least rifampicin with or without isoniazid resistance [1]. Clofazimine, a rhimophenazine dye, is classified by the World Health Organization (WHO) as a group B antituberculosis medication due to its independent association with successful MDR/RR-TB treatment outcomes in adults [2]. Clofazimine is included in current WHO-recommended regimens for children <15 years of age [3]. However, the pharmacokinetic and safety data for clofazimine are limited, even among adults treated for leprosy, the primary indication for this medication until a decade ago. The optimal clofazimine exposure for adults requiring treatment for MDR/RR-TB has not been well established due to a lack of pharmacokinetic-pharmacodynamic studies.

The efficacy of antituberculosis drugs and regimens in general is typically extrapolated from adult efficacy data, but the dosing and safety of these drugs in children need to be studied, with the most appropriate formulations. Although the Food and Drug Administration allows for a maximum daily clofazimine dose of 200 mg, the WHO-recommended daily dose of clofazimine is 2 to 5 mg/kg, with a maximum dose of 100 mg [4, 5]; therefore, adults weighing >50 kg are exposed to relatively lower doses (milligrams/kilograms) than most children. Access to dispersible clofazimine formulations for children is currently limited, but clofazimine is otherwise available in nondivisible 50- or 100-mg gel capsules. In settings where 50-mg formulations are unavailable, administration of accurate daily or alternate-day dosing in patients <20 kg (requiring <100 mg daily) is not feasible, and children are administered even higher doses (milligrams/kilograms) but at longer intervals (ie, alternate days or 2 or 3 times a week) to approximate the total weekly clofazimine dose thought to be required in adults, based on clinical experience and expert opinion only. There are limited published data to confirm the safety and exposure of current clofazimine dosing in children for tuberculosis with the existing gel capsule formulations. We aimed to describe the pharmacokinetics, tolerability, and safety of currently recommended doses of clofazimine in children routinely treated for MDR/RR-TB using 50-mg soft gel capsules (Novartis Pharma AG).

## METHODS

### Study Design, Setting, and Patient Population

This open-label, single-arm, phase 1/2 trial was conducted among children aged 0 to <18 years, with or without HIV, who were routinely receiving MDR/RR-TB treatment for <16 weeks in Cape Town, South Africa, between September 2020 and April 2022. Children were enrolled in 2 groups based on weight to ensure adequate broad distribution: cohort 1 (5–14 kg) and cohort 2 (≥15 kg). Only 100-mg clofazimine soft gel capsules (Novartis Pharma AG) were available in routine care at the time of this study.

Participants remained on study follow-up until completion of MDR/RR-TB treatment or for a maximum of 48 weeks, whichever came first. Target enrollment was 36 participants, with planned interim analysis and enrollment pause after 20 children had been enrolled, including a minimum of 6 participants in each cohort.

### MDR/RR-TB and HIV Treatment

Clofazimine 50-mg soft gel capsules (Novartis Pharma AG) were prescribed by weight (Table 1) in line with WHO weight-banded dosing recommendations [4] and administered by caregivers throughout the study. Participants received the rest of their MDR/RR-TB treatment through the local tuberculosis program and were treated according to local guidelines. Children receiving treatment in routine care prior to study entry switched from the routine 100-mg clofazimine capsules to the 50-mg study formulation shortly after enrollment. Children with HIV were enrolled regardless of antiretroviral therapy regimen.

**Table 1.** Study Clofazimine Doses with Observed Exposures and Proposed Dosing Adjustments with Predicted Exposures

Weight, kg	Daily Clofazimine Dose, mg	Actual Dose and Frequency, mg	Weekly AUC, mg × h/L, Median (IQR)
Study clofazimine weight-banded dosing with 50-mg capsules and observed weekly AUC^a^			
5 to <10	25^b^	50 (1 capsule) every second day	176.5 (128.1–208.2)
10 to <20	50	50 (1 capsule) daily	257.7 (201.3–271.0)
≥20	100	100 (2 capsules) daily	152.6 (122.4–215.4)
Proposed clofazimine dosing adjustments with 50-mg capsules and predicted weekly AUC^c^			
5 to <10	∼20^b^	50 (1 capsule) 3/wk	132.35 (96.08–156.18)
10 to <20	∼20^b^	50 (1 capsule) 3/wk	110.45 (86.27–116.16)
20 to <30	50	50 (1 capsule) daily	125.45 (86.55–164.35)
≥30	100	100 (2 capsules) daily	152.60 (130.20–208.30)

Abbreviations: AUC, area under the concentration time curve; IQR, interquartile range; WHO, World Health Organization.

^a^Study clofazimine doses with 50-mg gel capsule formulation, in line with WHO-recommended dosing for children [4] and observed weekly exposures at steady state.

^b^Average daily dose calculated by total weekly dose divided by 7.

^c^Proposed clofazimine dose adjustments with 50-mg gel capsule formulation to achieve modeled pediatric exposures closer to adult target exposures.

### Pharmacokinetic Sampling and Analysis

Baseline sparse pharmacokinetic sampling (at 2–6 hours postdose and immediately prior to the following dose) was carried out among children receiving the 100-mg clofazimine capsule prior to switching to the 50-mg capsule. Semi-intensive pharmacokinetic sampling was conducted at weeks 2 and 16 after initiation of the study clofazimine and included predose (0 hour) and 1-, 2-, 4-, 8-, and 24-hour samples after clofazimine administration. An additional 48-hour sample was collected from children who were dosed every second day. Children fasted overnight prior to sampling days. On the day of sampling, study personnel observed and recorded all food intake and administration of medications on-site. Study clofazimine was administered orally, either swallowed whole or softened and eaten in yogurt, within 30 minutes after a locally available fat-containing meal. Antiretroviral therapy and other MDR/RR-TB medications were administered ≥5 minutes after study clofazimine administration. In participants who had completed their MDR/RR-TB treatment within 48 weeks after enrollment, 2 further sparse samples were collected at 3 to 7 days and 14 to 28 days after completion of clofazimine. Planned interim pharmacokinetic and safety analysis was completed after 20 children (minimum 6 in each cohort) had successfully completed week 2 semi-intensive pharmacokinetic sampling, to review prespecified pharmacokinetic and safety targets and potential dose adjustments.

Plasma clofazimine concentrations were determined by a validated liquid chromatography–tandem mass spectrometry method performed according to standard operating procedures at a laboratory certified in good laboratory practice and accredited per ISO 15189 (FARMOVS [Pty] Ltd).

### Pharmacokinetic Targets

The original pharmacokinetic exposure target when the study was designed was based on the median area under the concentration-time curve (AUC) in adults receiving 100-mg clofazimine daily for the treatment of leprosy: daily AUC, 15.97 mg × h/L (IQR, 11.7–21.7); weekly AUC (wAUC), 111.79 mg × h/L (IQR, 81.9–151.9). During the study, published data reporting a wAUC of 60.87 mg × h/L (IQR, 56.00–66.64) among adults receiving clofazimine 100 mg daily for treatment of MDR/RR-TB [6] became available, which were considered relevant to our analysis. Therefore, weekly clofazimine exposures were also compared with this target. For planned interim analysis, the original protocol-specified pharmacokinetic target was defined as being met if the median AUC of clofazimine for each cohort of children was not more than 25% higher or 25% lower than the protocol-specified adult target.

### Pharmacokinetic Analysis

Clofazimine concentration data were analyzed by nonlinear mixed effects modeling. Population pharmacokinetic parameters were estimated with first-order conditional estimation with interaction. Interindividual and interoccasion variabilities were explored and modeled exponentially assuming a log-normal distribution, and 1, 2, and additional compartment disposition models were evaluated. First-order and nonlinear eliminations were evaluated. First-order absorption or absorption delay was evaluated. Selection between models was mainly based on the minimum value of the objective function provided by NONMEM, which is equal to −2 log likelihood with differences of 3.84, 7.88, and 10.83 being considered significant at the 5%, 0.5%, and 0.1% levels, respectively, for nested models differing in 1 parameter. Model building and evaluation were guided by goodness-of-fit plots, objective function value, and simulation-based diagnostics, such as visual predictive checks. NONMEM 7.5 and Pearl-speaks-NONMEM 4.7.0 were used for modeling and simulation. Visual diagnostics were done with the xpose (version 0.4.4) and vpc (version 1.0.1) packages in R. Primary pharmacokinetic parameters and other parameters of interest, such as weekly AUC exposures at steady state, were generated as outputs from the modeling.

Stepwise covariate modeling (*P* < .05 forward selection, *P* < .01 backward deletion) was performed to identify predictors of primary pharmacokinetic parameters. Evaluated covariates were body size, age, nutritional status (*Z* scores; weight, height, and body mass index for age), HIV status, and sex. Fat-free mass was calculated by the weight, height, and sex of the participants.

### Simulations to Inform More Optimal Dosing Strategies

Simulations were performed to derive alternative model-informed dosing schedules of clofazimine for children, based on the emerging target exposures in adults with MDR/RR-TB. Empirical Bayes estimates of model parameters were used to estimate AUC at steady state for individuals, with trial doses and under model-informed dosing. Weekly steady state AUCs were calculated to compare different dosing frequencies with the clofazimine 50-mg gel capsule formulation.

### Safety Monitoring and Assessment

Safety was monitored through clinical and laboratory assessments at monthly visits and when clinically indicated. Electrocardiograms (ECGs) were done in triplicate at each study visit and at 3 time points (predose and 4 and 8 hours postdose) on semi-intensive pharmacokinetic sampling days. All ECGs were evaluated by a pediatric cardiologist, and the QT interval was corrected for heart rate with the Fridericia formula (QTcF). Adverse events were assessed for attribution to clofazimine, and severity was graded by the DAIDS Adverse Events Grading Table [7]. The safety target was defined as being met if there were no more than 4 grade ≥3 adverse events overall that were at least possibly related to clofazimine.

QTcF interval data were modeled sequentially with a population approach in NONMEM where individual pharmacokinetic parameters were used to generate plasma concentrations at the time of each QTcF measure. The population baseline QTcF and interindividual variability were estimated by predose QTcF measures during clofazimine treatment, since true baseline QTcF without clofazimine therapy was not available. The effect of clofazimine was estimated during treatment. Age, sex, and use of concomitant QT-prolonging agents were tested as covariates on baseline and drug effect parameters stepwise, as described previously.

### Statistical Analysis

Clinical and demographic characteristics were summarized by descriptive statistics, and safety outcomes were reported in frequencies and proportions, all stratified by cohort. Continuous variables were reported with medians and IQRs while categorical variables were reported by frequencies and percentages. Weight- and height-for-age *Z* scores were calculated per British 1990 growth curves [8]. Adverse events related to clofazimine were displayed by grade via frequencies. Data were analyzed by Stata Special Edition 16.0 (StataCorp LP).

### Ethical Considerations

Written informed consent was provided by parents/legal guardians and written informed assent by participants aged ≥7 years. The study was approved by the Health Research Ethics Committee of Stellenbosch University, the South African Health Products Regulatory Authority, the Western Cape Government Department of Health and Wellness, and the City of Cape Town Health Department. The study was registered on the South African National Clinical Trials Register (DOH-27-0620-6415) on 30 October 2019.

## RESULTS

Twenty-two children were enrolled. Two children withdrew consent prior to week 2 semi-intensive pharmacokinetic sampling and were withdrawn from study. Twenty evaluable participants (10 children in cohort 1 and 10 in cohort 2) with a median age of 6.0 years (IQR, 1.6–14.4) were included in analyses (Table 2). All 10 children in cohort 2 weighed ≥20 kg at the time of enrollment and received 100-mg study clofazimine daily.

**Table 2.** Demographic and Clinical Characteristics of Children With Rifampicin/Multidrug-Resistant Tuberculosis Receiving Clofazimine

	Participants, No. (%) or Median (IQR)		
Characteristic	All (n = 20)	Cohort 1 (n = 10)	Cohort 2 (n = 10)
Male sex	6 (30.0)	3 (30.0)	3 (30.0)
Age, y	6.0 (1.6–14.4)	1.6 (0.7–2.1)	14.4 (13.6–15.8)
Weight, kg	19.1 (9.5–41.8)	9.5 (8.4–11.5)	41.8 (31.6–46.1)
Height, cm	110.7 (75.5–158.3)	75.5 (67.0–79.0)	158.3 (150.5–162.0)
*Z* score			
Weight for age	−1.27 (−2.21, −0.50)	−1.31 (−1.74, −0.54)	−0.91 (−2.42, −0.47)
Height for age	−1.57 (−2.48, −0.26)	−1.98 (−3.16, −1.64)	−0.51 (−1.45, 0.36)
TB classification			
Confirmed	16 (80.0)	6 (60.0)	10 (100.0)
Clinically diagnosed/unconfirmed	4 (20.0)	4 (40.0)	0 (0.0)
TB disease type			
PTB only	18 (90.0)	8 (80.0)	10 (100.0)
EPTB only	0 (0.0)	0 (0.0)	0 (0.0)
PTB and EPTB	2 (10.0)^a^	2 (20.0)^a^	0 (0.0)
Time undergoing, wk			
Routine CFZ formulation before study drug	9.0 (4.7–12.3)	5.4 (3.9–16.7)	10.0 (7.9–12.0)
Study CFZ formulation^b^	29.5 (29.2–34.7)	29.5 (28.0–34.0)	31.0 (20.1–39.4)
Any CFZ formulation^b^	39.1 (33.6–41.4)	39.1 (35.0–39.3)	39.6 (32.3–51.1)
Receipt of other QT-prolonging drugs			
Levofloxacin	18 (90.0)	10 (100.0)	8 (80.0)
Bedaquiline	10 (50.0)	0 (0.0)	10 (100.0)
Delamanid	3 (15.0)	1 (10.0)	2 (20.0)
HIV positive	2 (10.0)	1 (10.0)	1 (10.0)
Antiretroviral therapy regimen			
ABC-3TC-RTV-LPV	1 (50.0)	1 (100.0)	0 (0.0)
ABC-3TC-RTV-ATZ	1 (50.0)	0 (0.0)	1 (100.0)

Cohort 1: participants 5 to <15 kg. Cohort 2: participants ≥15 kg.

Abbreviations: 3TC, lamivudine; ABC, abacavir; ATZ, atazanavir; CFZ, clofazimine; EPTB, extrapulmonary tuberculosis; LPV, lopinavir; PTB, pulmonary tuberculosis; RTV, ritonavir.

^a^Both participants had abdominal and pulmonary tuberculosis.

^b^At the time of interim safety analysis.

Pharmacokinetic analysis was completed on all available samples when the 20th participant completed week 2 semi-intensive sampling. Of 20 evaluable participants, 14 (70%) also contributed data from semi-intensive pharmacokinetic sampling at week 16, while further sparse data were available for 7 (35%) participants following completion of clofazimine treatment.

### Pharmacokinetic Modeling

**Figure 1.**
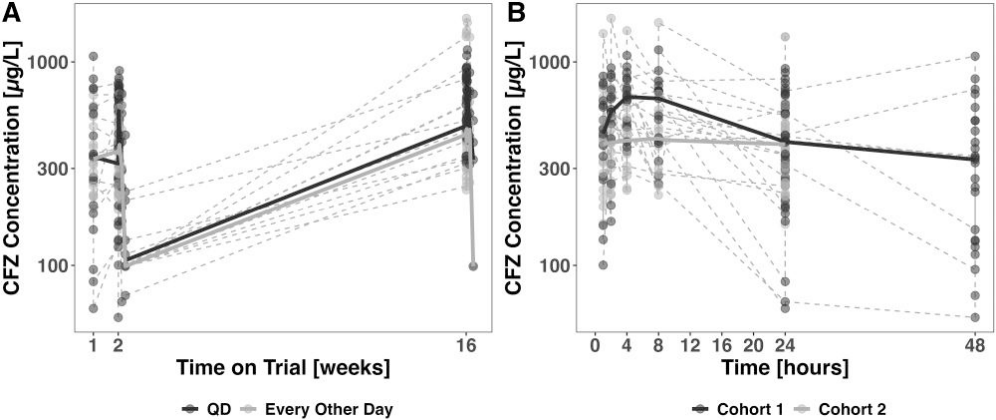
Raw concentrations of clofazimine reported by (*A*) time on trial and (*B*) time after dose, split by cohort. CFZ, clofazimine; QD, every day.

Raw clofazimine concentrations by cohort are demonstrated in Figure 1. Clofazimine concentration vs time profiles were best fit with a 2-compartment distribution model with first-order absorption and linear elimination. Allometrically scaling clearance (CL/F) and volume (V/F) by the mean population weight (24.9 kg) with exponents of 0.75 for clearance and 1 for volume significantly improved model fit. Similarly, interindividual variability was included on CL/F, resulting in a significant drop in objective function value. In addition, interoccasion variability between the first semi-intensive sampling occasion and the rest of the sampling occasions was included on F, improving the model fit. Body weight was a better covariate in the model than fat-free mass. The final parameter estimates are shown in Table 3. The visual predictive checks (Supplementary Figures 1–3) of the final model demonstrate good predictability of clofazimine concentrations overall as well as across weights and by frequency of clofazimine dosing.

**Table 3.** Frequency of Adverse Events Among Children With Rifampicin/Multidrug-Resistant Tuberculosis at Least Possibly Related to Clofazimine

		No. of Events by Grade				
Organ System Classification: Adverse Event Term	No. of Participants With Event	1	2	3	4	Total
Eye disorder						
Decreased visual acuity	1	0	1	0	0	1
Discolored sclerae	2	2	0	0	0	2
Gastrointestinal disorder						
Nausea, intermittent	2	3	0	0	0	3
Vomiting	1	1	0	0	0	1
Skin disorder						
Skin hyperpigmentation	18	11	7	0	0	18
Dry skin	7	8	1	0	0	9
Ichthyosis	5	3	2	0	0	5
Papular rash	2	2	0	0	0	2
Pruritic rash	1	1	0	0	0	1
Pruritis	1	1	0	0	0	1
Hepatobiliary disorder						
Elevated ALT	3	2	3	0	0	5
Drug induced liver injury	1	0	1	1	0	2
Cardiac disorder						
Prolonged QTcF	5	7	0	0	0	7
Other disorder						
Poor appetite	1	1	0	0	0	1
Hyperpigmented spots on dorsum of tongue	1	0	1	0	0	1

Abbreviations: ALT, alanine transferase; QTcF, QT interval corrected for heart rate per Fridericia formula.

**Figure 2.**
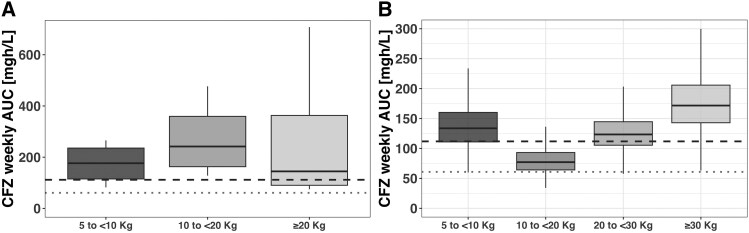
*A*, Comparison of observed clofazimine exposures for each weight-banded dosing group of children receiving clofazimine 50-mg gel capsules for treatment of rifampicin-resistant tuberculosis. *B*, Comparison of modeled predicted clofazimine exposures for the proposed amended dosing schedule with clofazimine 50-mg gel capsules. See also Table 1 for observed and predicted exposures. Bold dashed line: initial protocol-specified target exposure among adults receiving 100 mg of daily clofazimine for treatment of leprosy; median weekly AUC, 111.79 mg × h/L (IQR, 81.9–151.9). Lighter dotted line: subsequently published exposure among adults receiving 100 mg of daily clofazimine for treatment of RR-TB; median weekly AUC, 60.87 mg × h/L (IQR, 56.00–66.64). Data are otherwise presented as median (solid line), IQR (box), 95% CI (error bars). AUC, area under the concentration-time curve; CFZ, clofazimine; RR-TB, rifampicin-resistant tuberculosis.

The median clofazimine wAUC was 162.94 mg × h/L (IQR, 130.06–263.95). Among participants, the median wAUC was 176 mg × h/L (IQR, 128–236) in cohort 1 (5–14 kg) and 145 mg × h/L (IQR, 90.4–363) in cohort 2 (≥15 kg). Table 1 and Figure 2 demonstrate the median wAUC among participants by weight band. The initial protocol-specified pharmacokinetic target (wAUC, 111.79 mg × h/L) was therefore exceeded, as the median wAUC across both cohorts was >25% higher than the exposure among adults with leprosy. It was also considerably higher than recently published data from adults with MDR/RR-TB receiving clofazimine (wAUC, 60.87 mg × h/L).

### Safety

Overall, 212 adverse events were documented among all 20 participants; there were no life-threatening events. Three adverse events were considered serious but not related to the study drug, and 9 adverse events were graded as severe (only 1 possibly related to clofazimine). Fifty-nine adverse events (23%; 18 in cohort 1 and 41 in cohort 2) among 19 of the 20 participants were considered at least possibly related to clofazimine (Table 3, Supplementary Table 1).

One 14-year-old girl who was HIV negative had grade 3 elevated serum alanine transferase 6 months after starting the study drug. She was asymptomatic with no clinical signs of liver injury. All antituberculosis treatment (levofloxacin, terizidone, clofazimine) was temporarily withheld for 1 week. Alanine transferase decreased to grade 1; levofloxacin and terizidone were reintroduced; and all liver function test results returned to normal. Alanine transferase increased to grade 2 following reintroduction of clofazimine and continued to fluctuate over the next 3 months while the child continued treatment. She remained asymptomatic but was eventually withdrawn from the study 1 month later, when her liver function test results were in the normal range, due to overall poor adherence to MDR/RR-TB treatment.

**Figure 3.**
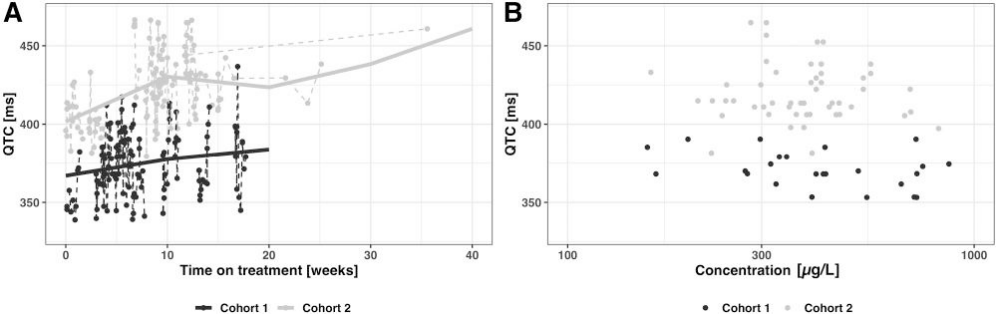
*A*, QTcF over time during clofazimine treatment by cohort. *B*, QTcF over clofazimine concentrations by cohort when paired samples were available. QTcF, QT interval corrected for heart rate per the Fridericia formula.

Seven episodes of QTcF prolongation (>460 to <480 milliseconds) occurred in 5 participants, all in cohort 2. The median QTcF at study enrollment was 368 milliseconds (IQR, 343–380) among cohort 1 and 431 milliseconds (IQR, 397–449) in cohort 2 (Figure 3). Five participants in cohort 1 experienced an increase in QTcF >30 milliseconds from screening as compared with 4 participants in cohort 2, while 3 in cohort 1 had an increase >60 milliseconds during the observed period vs 1 in cohort 2. No participants had QTcF ≥480 milliseconds recorded on ECG monitoring.

### Concentration-QTcF Relationship

Clofazimine-induced QTcF prolongation was best characterized with a direct linear concentration-response model. The investigation of an E_max_ model resulted in nonplausible estimates of the parameters and was therefore not carried forward. QTcF measurements at screening were used as baseline values. The model described the data well (Supplementary Figure 4). Parameter estimates are depicted in Table 4.

**Table 4.** Population Pharmacokinetic and Fridericia-Corrected QT Parameter Estimates in Children Treated With Clofazimine for Rifampicin/Multidrug-Resistant Tuberculosis

		Variability (CV %)	
Variable	Population Estimate (RSE %)	Interindividual	Interoccasion
Pharmacokinetic model			
CL/F, L/h^a^	3.07 (28)	58.5 (27)	
V/F, L^a^	282 (47)	…	…
Ka, 1/h	0.06 (37)	…	…
Q/F, L/h^a^	6.62 (14)	…	…
VP/F, L^a^	9450 (41)	…	…
F	1 fix	…	30.2 (22)
Residual error: additive, µg/L	185 (11)	…	…
Fridericia-corrected QT model			
Slope, ms/µg/L	0.02 (17)	…	…
Residual error: additive, ms	17.8 (5)	…	…

Abbreviations: CL/F, oral clearance; CV, coefficient of variation; F, bioavailability; Ka, absorption rate constant; Q/F, intercompartmental clearance; RSE, residual standard error; V/F, volume of distribution of the central compartment; VP/F, volume of distribution of the peripheral compartment.

^a^Scaled to mean body weight (24.9 kg), with exponents of 0.75 for CL and Q and 1 for V and VP.

The linear relationship between clofazimine concentrations and QT interval estimated a 0.02-millisecond increase per 1-µg/L increase in clofazimine concentration (Figure 3). Individual and treatment characteristics, such as coadministration of other QT-prolonging medications (bedaquiline and delamanid), did not significantly affect the model.

### Clofazimine Dose Simulations

As clofazimine exposures measured in children exceeded adult MDR/RR-TB exposures, model-adjusted doses of clofazimine were proposed by the 50-mg capsule formulations and practically feasible dosing schedules. The proposed revised dosing schedule with model-predicted weekly AUCs is shown in Table 1 and Figure 2.

## DISCUSSION

We report findings of the pharmacokinetics and safety of clofazimine 50-mg soft gel capsules in children with MDR/RR-TB. These interim results showed an acceptable safety profile, with only 1 severe clofazimine-related adverse event. Clofazimine exposures (wAUC) among children with MDR/RR-TB were >25% higher than the protocol-specified AUC target among adults treated with 100 mg for leprosy and higher still than the median wAUC exposures among adults treated with the same dose for MDR/RR-TB [6]. Our findings are remarkably different from published data from a cohort of 54 children with MDR/RR-TB (5 with HIV) who were receiving daily or intermittent dosing of 100-mg clofazimine capsules [9]. The median wAUC of 79.1 mg × h/L reported in that study, which was also conducted in South Africa, was higher than the comparative adult target of 60.9 mg × h/L [6] but less than half the wAUC (162.9 mg × h/L) observed in our study. The previous study sampled participants opportunistically for second-line antituberculosis drugs, and the median duration of clofazimine prior to sampling was 64 days, with only one-third of participants receiving daily clofazimine dosing [9]. Two-thirds of children in our study received daily dosing, with either 50- or 100-mg clofazimine, which might explain the higher exposures in our cohort. The effect of more frequent dosing with 50-mg capsules (1 or 2) as compared with intermittent dosing with 100-mg capsules on clofazimine exposures in younger children requires further evaluation.

Participant weight was identified as a covariate in our pharmacokinetic model and affected clearances and volumes of distribution. In contrast to the earlier study [9], there was no association between volume of distribution and age or any difference in drug clearance in children with HIV. This is not unexpected since our study included only 2 children with HIV; thus, numbers were too small to adequately assess the impact of HIV as a covariate on clofazimine pharmacokinetics in children. While adult females tend to have lower clofazimine concentrations than males due to higher body fat distribution [6], this difference is less pronounced in prepubertal children. Neither sex nor nutritional status affected the pharmacokinetic characteristics of clofazimine in our analysis.

The most concerning adverse effect of clofazimine is prolongation of the cardiac QT interval, which increases the risk of life-threatening arrhythmias [10] and is additive in conjunction with other QT-prolonging drugs [11]. Among adults with tuberculosis, 2 weeks of clofazimine dosed at 100 mg daily was associated with a QT interval increase of 16 to 20 milliseconds [12]. The relatively longer QT intervals among older children in our study were likely due to lower average heart rates and coadministration of bedaquiline, which was not included in the younger cohort's treatment regimens. No serious cardiac events were reported in either cohort. However, we described a linear relationship between clofazimine concentrations and QT interval prolongation with a drug effect of 0.02 ms/µg/L, which was independent of concomitant treatment with bedaquiline or delamanid. This clofazimine concentration–QT interval correlation is consistent with an even larger effect of 0.05 ms/µg/L in the other pediatric clofazimine study [9] and may be even more pronounced at a population level. In a modeling study to describe the relationship between clofazimine exposure and QT interval prolongation in adults with MDR/RR-TB, the estimated maximum change in QTcF was 28 milliseconds at steady state with standard clofazimine dosing, but the QT-prolonging effect of clofazimine was concentration driven [13]. In a multivariate analysis of 88 children receiving ≥1 QT-prolonging antituberculosis drugs for MDR/RR-TB, there was a low risk of QTcF interval prolongation in general, but clofazimine was associated with greater QTcF increases and maximum QTcF measurements when combined with fluoroquinolones as compared with other fluoroquinolone-based combinations [14]. In programmatic settings, where multiple QT-prolonging drugs are often recommended for treatment of MDR/RR-TB in children [15] and where regular ECG monitoring is challenging [16], unnecessarily high clofazimine exposures may pose a needlessly increased risk of QT interval prolongation in children.

Results presented here were from an interim analysis; therefore, the main limitation was the small sample size. The dosing amendments for the revised weight bands were based on data from just 2 or 3 children per weight band, but these amendments were proposed for further study in more children. Suboptimal adherence to dosing requirements is another potential limitation of this study, as prescribed doses of study medication were observed by the participants’ caregivers at the time of administration, but direct observation of the caregiver administering the medication was not specifically required. A further limitation is the lack of predrug baseline QTcF measurements, as most children in our study had already initiated MDR/RR-TB treatment, including clofazimine, in routine care prior to enrollment. Due to the linear relationship of QTc interval prolongation with drug concentration, our model may thus have underestimated the magnitude of the effect of clofazimine on QT interval.

Longer-term pharmacokinetic data were not available for every participant beyond the week 2 semi-intensive sampling visit, as these were not necessary for the planned interim analysis. Our proposed clofazimine dosing adjustments for the remainder of the study were based on pharmacokinetic modeling of available data at the time of interim analysis, but the study will not be completed as originally planned due to a lack of funding. Restricted access to child-friendly dispersible formulations necessitates intermittent dosing with nondispersible 50- and 100-mg capsule formulations, and pharmacokinetic and safety evaluation of such formulations remains important. Even though the predicted pediatric exposures at the adjusted clofazimine doses remain higher than the protocol-specified adult targets, lowering clofazimine doses even further to reduce exposures in children requires intermittent dosing schedules that are pragmatically difficult to deliver. The pharmacokinetic and safety evaluation of dispersible tablet formulations that allow daily dosing remains a priority. Future studies with dosing strategies informed by pooled data should evaluate lower doses and daily dosing strategies of different clofazimine formulations in children. Such work should also consider the use of newly proposed harmonized pediatric weight bands across disease areas, which may be adopted by the WHO in future.

## Supplementary Material

jiaf057_Supplementary_Data

## Supplementary Data

Supplementary materials are available at *The Journal of Infectious Diseases* online (http://jid.oxfordjournals.org/). Supplementary materials consist of data provided by the author that are published to benefit the reader. The posted materials are not copyedited. The contents of all supplementary data are the sole responsibility of the authors. Questions or messages regarding errors should be addressed to the author.

## Notes

***Acknowledgments.*** We thank the children and their families who participated in the study and the Desmond Tutu TB Centre study team members who implemented the study. We also acknowledge FARMOVS (Pty) Ltd, Bloemfontein, South Africa, for pharmacokinetic assays.

***Author contributions.*** E. N., J. A. H., I. C., and M. P. facilitated and coordinated the study. B. P. S. and R. M. S. carried out the pharmacokinetic analysis and interpretation of the data. J. C. N. assessed cardiac safety, and H. R. D. conducted the safety analysis. E. N., J. A. H., B. P. S., and H. R. D. developed the first draft of the manuscript. A. J. G.-P., H. S. S., L. v. d. L., and A. C. H. provided critical input for important intellectual content, and all authors approved the final version of the manuscript.

***Financial support.*** This study was partly funded by Novartis Pharma AG.

## References

[1] Jenkins HE, Yuen CM. The burden of multidrug-resistant tuberculosis in children. Int J Tuberc Lung Dis 2018; 22:3–6. 10.5588/ijtld.17.0357 PMC5975247 29665947

[2] World Health Organization . Rapid communication: key changes to treatment of multidrug- and rifampicin-resistant tuberculosis (MDR/RR-TB), August 2018. 2018. Available at: https://www.who.int/publications/i/item/WHO-CDS-TB-2018.18. Accessed 4 October 2023.

[3] World Health Organization . WHO consolidated guidelines on tuberculosis. Module 4: treatment—drug-resistant tuberculosis treatment, 2022 update. 2022. Available at: https://www.who.int/publications/i/item/9789240063129. Accessed 22 July 2023. 36630546

[4] World Health Organization . WHO operational handbook on tuberculosis. Module 4: drug-resistant tuberculosis treatment, 2022 update. 2022. Available at: https://www.who.int/publications/i/item/9789240065116. Accessed on 13 August 2024.

[5] World Health Organization . WHO consolidated guidelines on drug-resistant tuberculosis treatment. 2019. Available at: https://www.who.int/publications/i/item/9789241550529. Accessed 14 May 2024. 30946559

[6] Abdelwahab MT, Wasserman S, Brust JCM, et al Clofazimine pharmacokinetics in patients with TB: dosing implications. J Antimicrob Chemother 2020; 75:3269–77. 32747933 10.1093/jac/dkaa310 PMC7566350

[7] US Department of Health and Human Services , National Institutes of Health, National Institute of Allergy and Infectious Diseases, Division of AIDS. Division of AIDS (DAIDS) table for grading the severity of adult and pediatric adverse events, corrected version 2.1: July 2017. Available at: https://rsc.niaid.nih.gov/sites/default/files/daidsgradingcorrectedv21.pdf. Accessed on 7 January 2024.

[8] Cole TJ . Growth monitoring with the British 1990 growth reference. Arch Dis Child 1997; 76:47–9. 9059161 10.1136/adc.76.1.47 PMC1717048

[9] Ali AM, B PS, Hesseling AC, et al Pharmacokinetics and cardiac safety of clofazimine in children with rifampicin-resistant tuberculosis. Antimicrob Agents Chemother 2023;68:e0079423. 38112526 10.1128/aac.00794-23 PMC10777824

[10] Roden DM . Drug-induced prolongation of the QT interval. N Engl J Med 2004; 350:1013–22. 14999113 10.1056/NEJMra032426

[11] Wallis RS . Cardiac safety of extensively drug-resistant tuberculosis regimens including bedaquiline, delamanid and clofazimine. Eur Respir J 2016; 48:1526–7. 27799400 10.1183/13993003.01207-2016

[12] Diacon AH, Dawson R, von Groote-Bidlingmaier F, et al Bactericidal activity of pyrazinamide and clofazimine alone and in combinations with pretomanid and bedaquiline. Am J Respir Crit Care Med 2015; 191:943–53. 25622149 10.1164/rccm.201410-1801OC

[13] Abdelwahab MT, Court R, Everitt D, et al Effect of clofazimine concentration on QT prolongation in patients treated for tuberculosis. Antimicrob Agents Chemother 2021; 65:e0268720. 33875426 10.1128/AAC.02687-20 PMC8218646

[14] Ali AM, Radtke KK, Hesseling AC, et al QT interval prolongation with one or more QT-prolonging agents used as part of a multidrug regimen for rifampicin-resistant tuberculosis treatment: findings from two pediatric studies. Antimicrob Agents Chemother 2023; 67:e0144822. 37358463 10.1128/aac.01448-22 PMC10353402

[15] World Health Organization . WHO consolidated guidelines on tuberculosis. Module 5: management of tuberculosis in children and adolescents. 2022. Available at: https://www.who.int/publications/i/item/9789240046764. Accessed 22 July 2023. 35404556

[16] Nhassengo P, Zandamela A, Nhamuave C, et al Usability of simplified audiometry and electrocardiogram during treatment of drug-resistant tuberculosis in Mozambique: a qualitative study. BMC Global Public Health 2024; 2:12. 39681904 10.1186/s44263-024-00039-4 PMC11622995

