# Supplementary material for: Pharmacokinetics and Safety of Clofazimine in Children With Rifampicin-Resistant Tuberculosis
Source: J Infect Dis. 2025 Feb 11;231(5):e873–81. doi: 10.1093/infdis/jiaf057 (PMC12128054; doi:10.1093/infdis/jiaf057)

**SUPPLEMENTARY MATERIALS for manuscript number JID-80624R1:**

Pharmacokinetics and safety of clofazimine in children with rifampicin-resistant tuberculosis

**Table S1. Summary of frequency of adverse events at least possibly related to clofazimine, by cohort, among children with rifampicin-resistant/multidrug-resistant tuberculosis.**

|  | All Participants  (n=20) | Cohort 1  (n=10) | Cohort 2  (n=10) |
| --- | --- | --- | --- |
| Number of related AEs | 59 | 18 | 41 |
| Number of related Grade 1 (Mild) AEs | 42 | 14 | 28 |
| Number of related Grade 2 (Moderate) AEs | 16 | 4 | 12 |
| Number of related Grade 3 (Severe) AEs | 1 | 0 | 1 |
| Number of related Grade 4 (Life-threatening) AEs | 0 | 0 | 0 |

Cohort 1: <15.0 kg; Cohort 2: ≥15.0 kg

AE: adverse event

**Figure S1. Visual predictive check of interim analysis model of clofazimine (CFZ) concentration over time after dose in hours. The solid and dashed lines represent the median, 5^th^ and 95^th^ percentile of observed clofazimine (CFZ) concentrations, respectively. The shaded areas represent the prediction intervals generated by the popPK model for the median, 5^th^ and 95^th^ percentiles, respectively.**


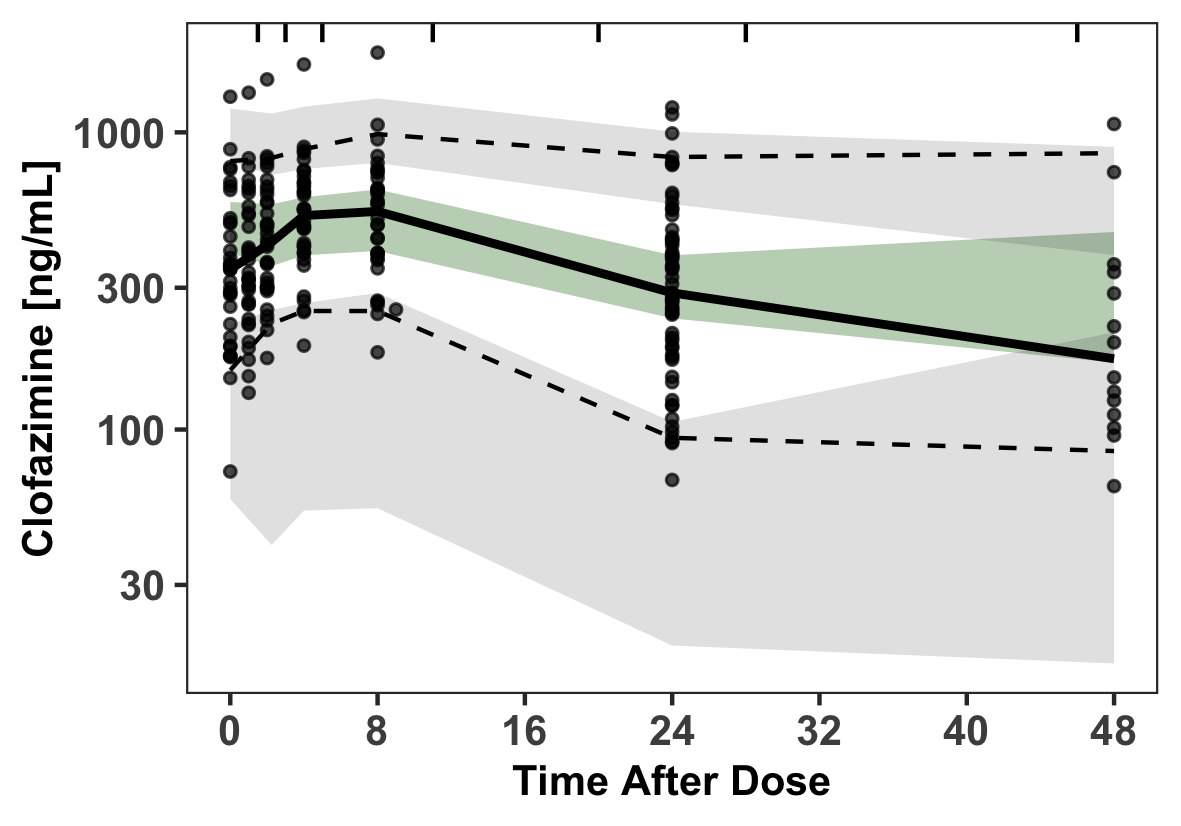


**Figure S2. Visual predictive check of analysis model of clofazimine concentration by time after dose in hours split by cohort. The solid and dashed lines represent the median, 5^th^ and 95^th^ percentile of observed clofazimine (CFZ) concentrations, respectively. The shaded areas represent the prediction intervals generated by the popPK model for the median, 5^th^ and 95^th^ percentiles, respectively.**


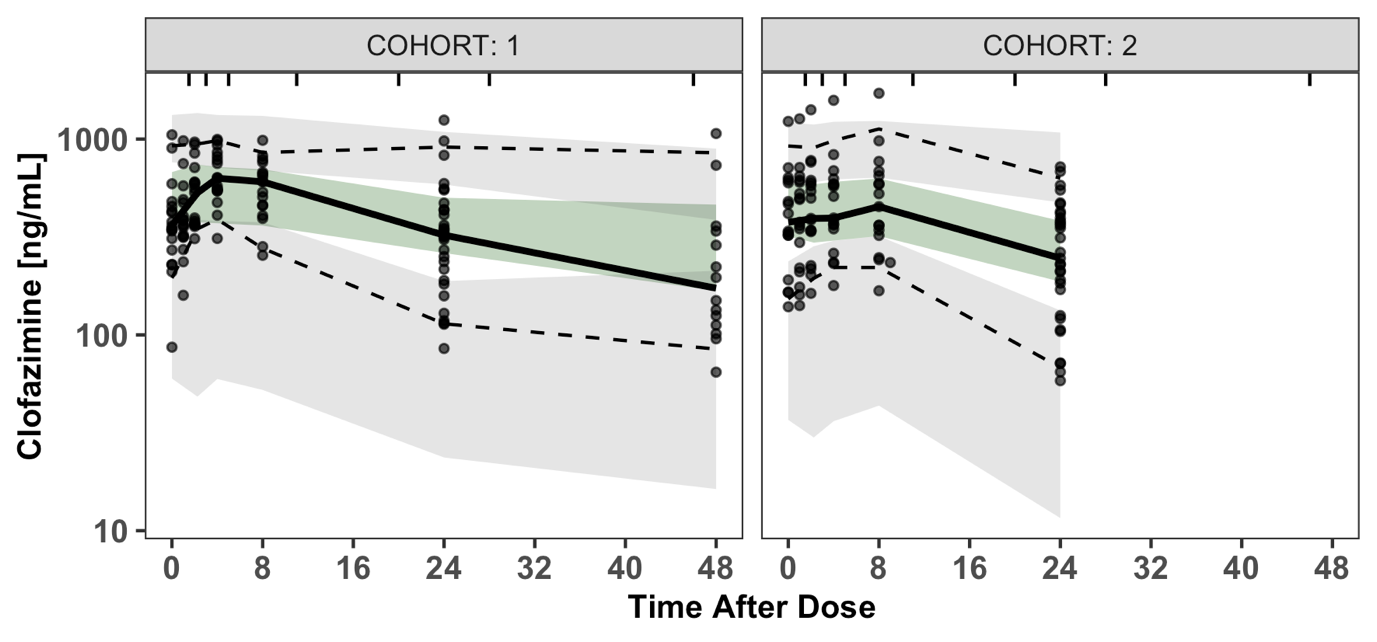


**Figure S3. Visual predictive check of interim analysis model of clofazimine concentration by time after dose in hours split by dosing frequency (every day [QD] and every other day [QOD]). The solid and dashed lines represent the median, 5^th^ and 95^th^ percentile of observed clofazimine (CFZ) concentrations, respectively. The shaded areas represent the prediction intervals generated by the popPK model for the median, 5^th^ and 95^th^ percentiles, respectively.**


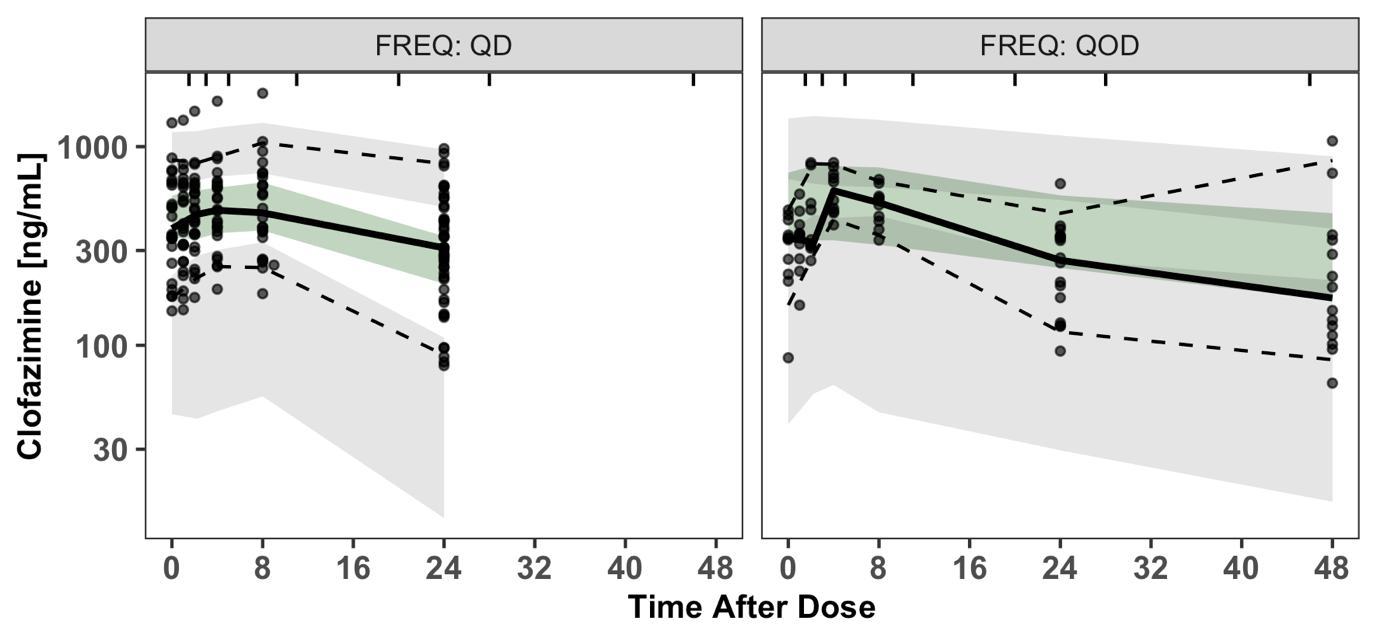


**Figure S4. Visual predictive check of clofazimine concentration-QTcF prolongation model over time after dose in hours. The solid and dashed lines represent the median, 5^th^ and 95^th^ percentile of observed QTcF, respectively. The shaded areas represent the prediction intervals generated by the PKPD model for the median, 5^th^ and 95^th^ percentiles, respectively.**


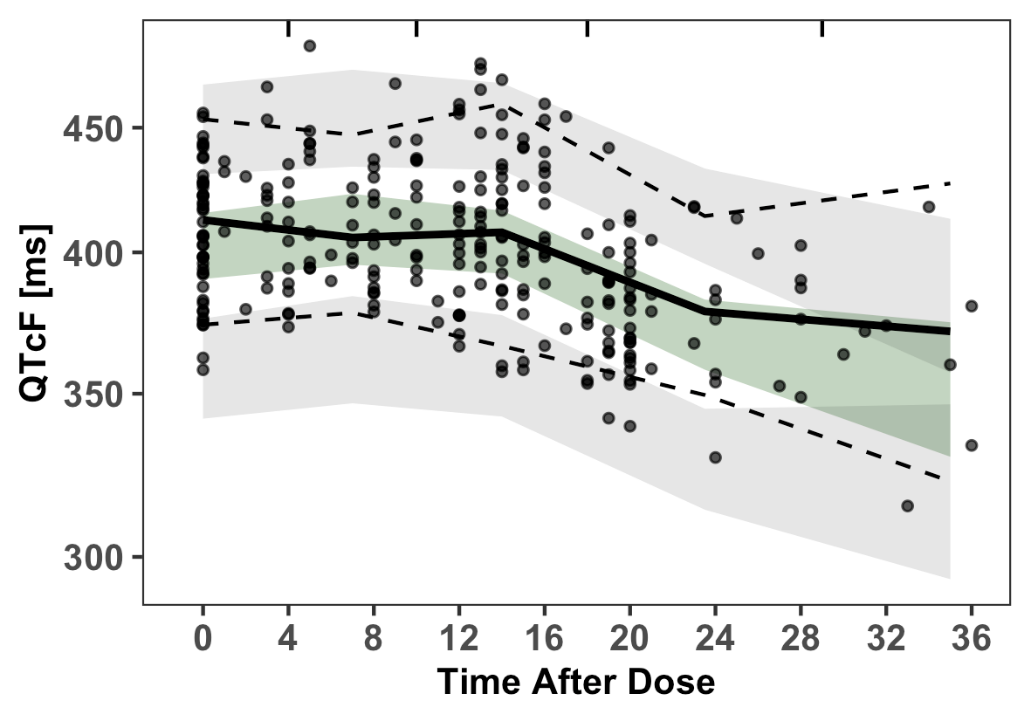

Supplement: jiaf057_Supplementary_Data [file jiaf057_supplementary_data.docx]
